# Technological Advances in SPECT and SPECT/CT Imaging

**DOI:** 10.3390/diagnostics14131431

**Published:** 2024-07-04

**Authors:** Yassine Bouchareb, Afrah AlSaadi, Jawa Zabah, Anjali Jain, Aziza Al-Jabri, Peter Phiri, Jian Qing Shi, Gayathri Delanerolle, Srinivasa Rao Sirasanagandla

**Affiliations:** 1 Department of Radiology & Molecular Imaging, College of Medicine and Health Sciences, Sultan Qaboos University, Muscat 123, Oman; 2 Department of Radiology & Molecular Imaging, Sultan Qaboos University Hospital, Muscat 123, Oman; 3 Sultan Qaboos Comprehensive Cancer Care and Research Centre, Department of Radiology, Muscat 123, Oman; 4 Southern Health NHS Foundation Trust, Southampton SO40 2RZ, UK; 5 Psychology Department, Faculty of Environmental and Life Sciences, University of Southampton, Southampton SO17 1BJ, UK; 6 Southern University of Science and Technology, Southampton, UK; 7 Southern University of Science and Technology, Shenzhen 518055, China; 8 University of Birmingham, Birmingham, UK; 9 Department of Human & Clinical Anatomy, College of Medicine and Health Sciences, Sultan Qaboos University, Muscat 123, Oman

**Keywords:** SPECT/CT, SPECT, collimator design, solid-state detectors, reconstruction algorithms, artificial intelligence

## Abstract

Single photon emission tomography/computed tomography (SPECT/CT) is a mature imaging technology with a dynamic role in the diagnosis and monitoring of a wide array of diseases. This paper reviews the technological advances, clinical impact, and future directions of SPECT and SPECT/CT imaging. The focus of this review is on signal amplifier devices, detector materials, camera head and collimator designs, image reconstruction techniques, and quantitative methods. Bulky photomultiplier tubes (PMTs) are being replaced by position-sensitive PMTs (PSPMTs), avalanche photodiodes (APDs), and silicon PMs to achieve higher detection efficiency and improved energy resolution and spatial resolution. Most recently, new SPECT cameras have been designed for cardiac imaging. The new design involves using specialised collimators in conjunction with conventional sodium iodide detectors (NaI(Tl)) or an L-shaped camera head, which utilises semiconductor detector materials such as CdZnTe (CZT: cadmium–zinc–telluride). The clinical benefits of the new design include shorter scanning times, improved image quality, enhanced patient comfort, reduced claustrophobic effects, and decreased overall size, particularly in specialised clinical centres. These noticeable improvements are also attributed to the implementation of resolution-recovery iterative reconstructions. Immense efforts have been made to establish SPECT and SPECT/CT imaging as quantitative tools by incorporating camera-specific modelling. Moreover, this review includes clinical examples in oncology, neurology, cardiology, musculoskeletal, and infection, demonstrating the impact of these advancements on clinical practice in radiology and molecular imaging departments.

## 1. Introduction

Photon emission computed tomography (SPECT) produces a three-dimensional (3D) distribution of the gamma rays emitted by radionuclides. The images provide functional information about organs and tissues, enabling the detection of functional abnormalities before anatomical changes occur. The combination of SPECT and computed tomography (CT) has enabled hybrid SPECT/CT scanners to be widely installed globally since 1999 [1]. SPECT/CT is a non-invasive hybrid technique that directly fuses morphological and functional information from the CT and SPECT components, respectively. CT images reveal the localisation of radionuclides and provide a means for attenuation correction of SPECT emission images [1,2]. SPECT/CT plays an increasingly important role in clinical practice, aiding diagnosis and assessment of the therapy response, particularly in cardiovascular diseases, cancer, and neurological disorders [1]. Several studies have demonstrated the added benefit of hybrid SPECT/CT compared with imaging modalities, including for the imaging of benign and malignant skeletal diseases, cancer of the thyroid, parathyroid, and pelvic regions, and sentinel lymph nodes [2,3,4]. Overall, SPECT/CT technology provides a more comprehensive imaging approach, improving diagnostic accuracy and localisation and enhancing reader confidence and clinical outcomes in several clinical applications, despite the ongoing advances in dedicated SPECT cameras [5,6].

Recent technological advances are strengthening the competitive position of SPECT/CT among diagnostic imaging techniques [3]. These advances encompass both the hardware used in data collection and software techniques that enable better manipulation of projection data to create high-quality images, making the subsequent image analysis and quantification of clinically useful parameters easier. The sophistication of SPECT and SPECT/CT hardware has led to the introduction of more sensitive detector materials, with scintillator NaI(Tl) detectors being replaced with semiconductor detectors [1,2]. Another notable development is the replacement of conventional photomultiplier tubes (PMTs) with signal amplifier devices such as position-sensitive PMTs (PSPMTs), avalanche photodiodes (APDs), and silicon PMs (SiPMs). Additionally, new designs of collimators and camera heads have improved the performance of SPECT and SPECT/CT systems in single-purpose and multi-purpose applications [1,3]. The development of software techniques in SPECT and SPECT/CT imaging was necessary to exploit the high photon-counting properties of the new hardware technologies [1]. The most noticeable improvements in software techniques include the implementation and commercial availability of reconstruction algorithms, data correction methods, and quantitative methods [3,4].

This paper reviews recent technological advancements in hardware and software tools and future directions in SPECT and SPECT/CT imaging, as well as their impact on clinical imaging, including in cardiology, oncology, musculoskeletal, neurology, and infectious and inflammatory diseases.

## 2. Advancements in SPECT Technology

**Figure 1:**
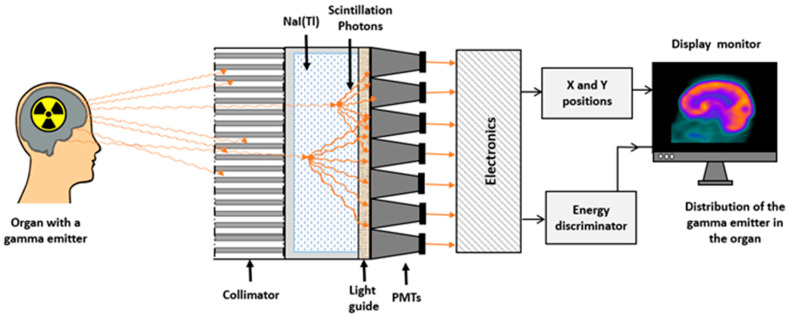
Schematic representation of basic components of the Anger scintillation camera.

The Anger scintillation cameras’ basic technological concepts used in SPECT imaging have not changed over the past 50 years [1,2]. Figure 1 shows a diagram of an Anger scintillation camera with its basic hardware components. Until the early years of this century, this design had several limitations, compromising its counting performance and increasing the delivered radiation doses and imaging time. Photomultiplier technology led to a large and bulky camera head that require extended spaces. They were also inconvenient for scanning immobile and paediatric patients [2,3]. The physical characteristics of NaI(Tl) crystals and PMTs have limited the photon energy resolution. Furthermore, the spatial resolution is reduced by the limited number of PMTs and the geometric characteristics of the collimators. The sensitivity of the Anger scintillation camera is relatively low due to several factors, including photon collimation, scintillation crystal efficiency, photon absorption and scatter, and energy window settings. Hence, longer acquisition times or higher doses of radiopharmaceuticals are needed to obtain clinically useful images [2].

Recently, there have been many advances in SPECT and SPECT/CT, including in both hardware components and software techniques [4].

Since the early years of the last decade, there has been widespread adoption of new technologies such as solid-state technology for both detectors (e.g., cadmium–zinc–telluride) and read-out systems, which replace photomultiplier tubes (avalanche photodiodes or silicon photomultipliers) [7]. The rapid growth and technical advances in SPECT and SPECT/CT are expected to continue, securing a more competitive position among diagnostic imaging techniques [3].

### 2.1. Advances in Hardware Technology

Current research focuses on exploring more a compact design for gamma cameras dedicated to small-organ imaging, myocardial perfusion imaging, scintimammography or molecular breast imaging, and small-animal imaging [2]. The smaller and more compact imaging systems are the product of significant improvements in the Anger scintillation camera hardware, including new detector materials and signal amplifying devices and improvements in the design of collimators and of the camera head [1,3,4]. 

#### 2.1.1. Detector Materials

The quality of the projection information required for SPECT depends on the physical properties of the γ-ray detectors. Detector material efficiency and material density are fundamentally tied to how effectively the detector material can interact with and detect the incoming radiation. Dense materials have high stopping power, which enhances the probability of detecting γ-rays and thus allows for accurate radiation measurements. On the other hand, low-density materials have low stopping power, reducing the chances of interaction and, consequently, lowering the detection efficiency, resulting in less accurate measurements. The conventional Anger scintillation camera has a large detector geometry with lower assembly costs and provides continuous sampling [3,8]. However, it has many limitations, especially for γ-rays with energy lower than 200 keV; the intrinsic efficiency is high, but the energy resolution and intrinsic spatial resolution are moderate.

Moreover, the position information is determined by Anger logic circuits, which degrade near the detector edge, resulting in several centimetres of “dead” space [7]. This is a significant concern for small field-of-view systems but is not a substantial problem for large detectors. Consequently, ongoing research aims to improve detector materials and head configurations. Advanced detectors aim to achieve high intrinsic efficiency by using materials with high atomic numbers (high density) [3,9]. Additionally, these detectors should be capable of offering better energy resolution and intrinsic spatial resolution, both of which depend on the strength of the signal generated from each event [7].

**Figure 2:**
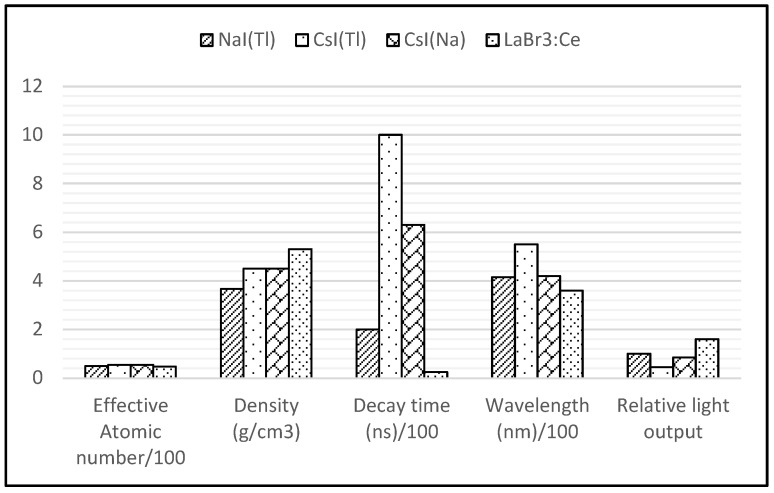
Main physical properties of the most commonly used scintillators in SPECT (the values, with the exception of density, are divided by 100 to scale the values to a similar range).

Along with the trend towards creating compact and preferred systems for small-field-of-view configurations (with non-dead edges), pixelated detectors have been integrated into gamma-camera designs [3,4]. Predominantly, these detectors utilise scintillating materials, which offer numerous advantages, including high intrinsic efficiency and low fabrication costs with various shapes and sizes [3,8,9]. The physical properties of the scintillators, namely NaI(Tl), CsI(Tl), CsI(Na), and LaBr3:Ce, are presented in Figure 2 [8]. These scintillating materials exhibit similar photopeak efficiencies due to their comparable effective atomic numbers and densities. The detectors’ counting rate capacity is limited by the scintillation’s persistence, while counting-rate losses with SPECT are rarely an issue when conventional collimation is employed.

The wavelength of the scintillation light determines the type of photon transducer that can be used to convert the scintillation light into an electronic pulse. The light output, relative to the scintillator, directly impacts the potential energy resolution of the system [3]. NaI(Tl) detectors have been utilised for decades in SPECT applications as a single large crystal [5]. However, they can also be pixelated. Pixelated NaI(Tl) scintillation detectors can be employed in configuring small-field-of-view devices (e.g., small animal SPECT systems) [8]. Thallium-activated caesium iodide, CsI(Tl), is not highly hygroscopic, and it competes favourably with NaI(Tl) with regards to efficiency [7]. It exhibits a relatively significantly longer scintillation time, leading to a larger dead time, although this is not a concern for SPECT [3,9].

The scintillation light from CsI(Tl) has a longer wavelength that is not as well matched to PMTs with NaI(Tl). As a result, the performance of CsI(Tl) with PMTs is worse than that of NaI(Tl), even though the total number of scintillation photons is about 18% higher than with NaI(Tl) [3]. CsI(Tl) detectors are suitable for use with photodiode signal amplifiers, enabling detection with high quantum efficiency. This approach is used by Digirad Corporation in their Cardius^®^ product [2]. Sodium-activated caesium iodide, CsI(Na), is similar to CsI(Tl), but its emission matches PMTs more closely. CsI(Na) detector material is utilised by LinoView in its small animal SPECT systems [9].

Lanthanum bromide (LaBr3) detector material possesses numerous attractive properties for time-of-flight PET and SPECT. In comparison with NaI(Tl), LaBr3 material detectors exhibit high light output, improved energy resolution (<6% at 140 keV), and intrinsic efficiency. Additionally, they are exceptionally fast detectors. Consequently, LaBr3 is a valuable material for high-counting rate detectors such as Compton γ-camera detectors. Moreover, LaBr3 is a preferred scintillation material for small-animal SPECT system detectors [3].

It is important to note that scintillators require an additional stage (photon transducer) to produce electronic pulses from emitted light. Consequently, light is lost at the interface between the detector and the photon transducer, limiting the energy resolution [3]. Recently, solid-state devices like cadmium–zinc–telluride (CZT) semiconductor detectors have become widely used in SPECT and SPECT/CT, especially for cardiac imaging applications [2,10]. CZT has the ability to directly convert the absorbed γ-ray energy into an electronic signal [7]. 

These devices are small and operate at low voltage since no intermediate high-gain amplification stage (like PMTs) is required [3]. Moreover, the energy resolution of semiconductor detectors is better than that of scintillator detectors because the produced signals are consistent, stable, and closely match and reflect the energy of the detected photons. CZT semiconductor detectors operate at room temperature. The CZT’s intrinsic efficiency is close to that of NaI(Tl), having similar thicknesses; hence, energy resolutions of 2–5% are easily attained at 140 keV γ-rays. Therefore, CZT is suitable for multi-radionuclide and kinetic studies [7]. Although considerably finer resolution can be achieved, CZT is accessible as pixelated detector arrays with an intrinsic spatial resolution of 2.4 mm.

The semiconductor detectors’ disadvantages are mainly their fabrication costs and their intrinsic efficiency for high-energy γ-rays [10]. However, over the past decade, this technology has seen significant improvements, leading to a reduction in production costs. Furthermore, it has been implemented in devices with a small field of view. D-SPECT^®^, VERITON^®^, and several small-animal SPECT systems are examples of applications utilising semiconductor devices [2,10].

#### 2.1.2. Signal Amplifying Devices

Photomultiplier tubes (PMTs) are traditionally employed for several decades for the conversion of scintillation light into electronic signals [3]. PMTs offer large electronic gain (10^6^) at a very low noise. Nevertheless, they have a several drawbacks. Firstly, their low (about 20%) quantum conversion efficiency results in a large signal loss that compromises both the inherent spatial resolution and the energy resolution [11,12]. It is difficult to maintain their properties for a long time because they are sensitive to environmental changes (temperature, humidity, and magnetic fields). Their performance also changes as they age. Photomultiplier Tubes (PMTs) are being replaced by position-sensitive PMTs (PSPMTs), avalanche photodiodes (APDs), and silicon photomultipliers (SiPMs) [11,13]. PMTs and solid-state photodiodes have comparable gain, timing resolution, and energy resolution. However, PMTs are bulky, expensive, and not MR compatible. 

In contrast to conventional PMTs, which lack intrinsic localisation, PSPMTs (a matrix of PMT elements) exhibit high position sensitivity and gain [3,14]. They are also more efficient and compactly designed. PSPMTs are suitable for pixelated detectors due to the wide gain variation across their field [15]. Similar to conventional PMTs, PSPMTs are influenced by environmental factors such as magnetic fields [3,16]. Additionally, PSPMTs have been utilised in small-field-of-view devices with individual pixelated detectors [14]. 

Solid-state read-out devices such as APDs and SiPMs are used instead of PMTs to convert light emitted from a scintillator into electrical signals. As these devices are not affected by magnetic fields like PMTs are, they are ideal for hybrid SPECT and PET imaging combined with MRI. They are also appropriate for uses where a small system design is necessary [11].

Avalanche photodiodes (APDs), compared with PMTs, are more rugged and compact and are highly immune to environmental factors [17]. They boast a high quantum conversion efficiency (>65%) and operate at lower voltages compared with PMTs. Additionally, currently available APDs have a maximum gain of about 250 [3]. They are well suited for pixelated detectors and for use with CsI(Tl) or other scintillators that emit light with longer wavelengths [18]. Silicon photomultipliers (SiPMs) are, at present, the only photodetectors well developed enough to be included in reliable, cost-effective, and commercial cameras [3,19]. SiPMs feature a compact design, are not affected by environmental factors, and exhibit greater than 50% quantum efficiency. Moreover, they offer high gains (10^5^–10^6^) with low bias levels (~30 V) [20].

#### 2.1.3. Design of Collimators

The statistical uncertainties in SPECT are minimised by employing a detector system with a large active area and an acquisition geometry that maximises the utilisation of the emitted gamma photons [21]. Additionally, the increasing interest in shortening the acquisition time or reducing the administered dose has spurred the development of new hardware and sophisticated data acquisition, reconstruction, and processing techniques, aiming to enhance the image quality and diagnostic accuracy to levels comparable to or even surpassing those of standard protocols [22,23].

Recently, IQ.SPECT technology has allowed ultra-fast cardiac imaging to be performed compared with general-purpose gamma cameras [22]. IQ.SPECT minimises statistical uncertainties and enables the selection of optimised protocol options. It also reduces the acquisition time and/or administered dose [24]. Moreover, IQ.SPECT is based on a unique collimator design (referred to as a SMARTZOOM collimator), modified cardio-centric acquisition, and special reconstruction methods [22,24]. IQ.SPECT maintains the same reconstructed resolution as conventional LEHR collimators in a non-circular orbit. It also corrects for patient attenuation effects, scatter, and the detectors’ motion relative to the centre of rotation of the gamma camera to avoid image artifacts and misalignment, ensuring that acquired images are sharp and correctly aligned [25].

**Figure 3:**
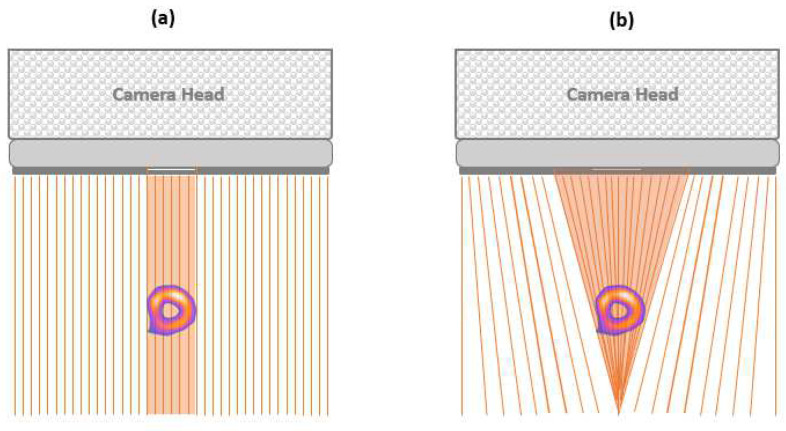
Camera head with a conventional LEHR collimator (**a**) and a SMARTZOOM collimator (**b**).

In the IQ.SPECT multifocal collimator, the holes are near-parallel at the edge and focused at the centre. Therefore, within the field of view, it mimics the properties of both cone-beam collimators (magnification at the centre) and parallel-hole collimators (eliminating truncation at the edges) [24,25]. Furthermore, it permits the capture of untruncated projection data while enabling an increase in sensitivity for the heart region [26,27]. The SMARTZOOM collimator presents 48,000 holes, each of which is referred to as a vector map. During image reconstruction, these holes are measured and stored within the collimator [22,25]. Hence, it enables the collection of up to four times more counts than conventional myocardial perfusion imaging (MPI), which has a low-energy high-resolution (LEHR) collimator, thereby providing four-fold higher sensitivity [24,28]. Figure 3 illustrates the differences in design between conventional (e.g., LEHR) and SMARTZOOM collimators.

Typically, the detectors’ cardio-centric orbit is placed on the heart rather than at the mechanical centre of the gantry. Consequently, the heart is positioned within the magnification area where the centre of the collimator field of view is located [25,28]. IQ.SPECT, as opposed to traditional cardiac SPECT systems, makes use of the camera gantry’s flexibility to place each detector at the ideal distance of a 28 cm radius for the cardio-centric orbit, which reduces claustrophobia and increases sensitivity [25]. Furthermore, in conjunction with sophisticated reconstruction, IQ.SPECT provides an array of optimal protocol alternatives, including a 4 min standard dose, an 8 min half-dose, and a 16 min quarter dose [22,29].

The size and form of the collimator hole, gantry deflections, detector motions, and the distance between the patient and the detectors are factors that are not fully taken into account by conventional reconstruction techniques [25,27]. On the other hand, the sophisticated IQ•SPECT reconstruction technique employs a conjugate-gradient iterative reconstruction algorithm that has been fine-tuned to precisely capture the observed geometry of the SMARTZOOM collimators and the detectors’ cardio-centric orbit [25]. Furthermore, it allows for correction of gantry motion-based image distortions by a 3D measurement of gantry deflection [24]. By determining the size, shape, and pointing direction of each of the 48,000 holes in the SMARTZOOM collimator, it also guarantees precise image reconstruction. As a result, IQ.SPECT minimises truncation and artifacts that might degrade image quality [26,30]. 

#### 2.1.4. Design of the Camera Head

Traditional dual-head gamma cameras are half a century old design-wise. However, because the heart is only imaged using a small percentage of the available NaI(Tl) crystal detector surface, it is inefficient for cardiac imaging [1]. Moreover, it increases the claustrophobic effects for patients due to the close distance between the detector and the patient. Therefore, new dedicated nuclear cardiology cameras have been developed by many manufacturers of SPECT cameras [1,10]. These dedicated SPECT cameras, such as D-SPECT, Discovery 530c, Cardius 3 XPO, and VERITON, have unique camera head designs. Furthermore, these cameras reduce radiation doses to patients or shorten the scanning time. They also promote easier scheduling, higher patient satisfaction, and, more importantly, higher image quality [10,31].

D-SPECT (Spectrum Dynamics) is the first clinical cadmium–zinc–telluride (CZT)-based nuclear cardiac imaging system [32,33]. Moreover, it is available with nine digital CZT-based detectors (D-SPECT™) and with six digital CZT-based detectors, offering a more cost-effective solution. D-SPECT improves the sensitivity and energy resolution dramatically [32]. Therefore, the detector sensitivity is up to ten times that of conventional cameras, allowing for a dramatic reduction in the injected dose [34]. It shortens the scanning time by a factor of 3–4 compared with conventional SPECT [32,34,35]. The exceptional energy resolution allows for simultaneous multiple energy studies [10,32]. With its open gantry design, D-SPECT may collect data without requiring the patient to have the detectors rotated around them from the left posterior oblique (LPO) to the right anterior oblique (RAO). This removes the possibility of pinch spots, claustrophobia, and acquisition collisions that may arise from moving detectors [33].

Discovery NM (GE Healthcare) systems are based on Alcyone technology, which consists of CZT detectors, focused collimation, 3D reconstruction, and stationary data acquisition [10,34]. Alcyone technology is available in SPECT configurations [35,36]. Furthermore, it offers up to four times the sensitivity without any equipment motion for improved image quality [36]. Cardius 3 XPO (Digirad) increases the nuclear cardiology efficiency with its triple-head system. The triple-head system increases patient throughput by up to 38% [37]. It is a high-definition system that uses solid-state detectors to enhance image quality [33]. Moreover, it is a flexible and compact system that can be installed in rooms as small as 7′ by 8′. Cardius 3 XPO enhances comfort for obese or claustrophobic patients, thus optimising the diagnosis process [37]. 

VERITON (Spectrum Dynamics) is a multi-purpose SPECT scanner with 12 independent CZT detector arms to provide 360-degree coverage, which is suitable for the three-dimensional human body [38]. Compared with conventional twin-head arrays, photon detection is maximised and the image quality is improved [39]. The VERITON system is uniquely designed to allow for quantitative 3D SPECT dynamic imaging with applications such as cardiac imaging, whole-body bone scans, and neuro-molecular imaging as an alternative to PET [38].

SPECT instrumentation will develop along two parallel tracks: multi-purpose and single-purpose systems [40]. Furthermore, ultrafast SPECT cameras with detector technology that incorporate silicon photodiodes and solid-state detectors such as CsI(Tl), CsI(Na), LaBr3:Ce, and CZT, are continuing to spread within specialised clinical centres [10]. Multi-purpose gamma cameras, which use semiconductor detector materials such as CZT, will surely receive special attention from many manufacturers of SPECT and SPECT/CT systems to reduce production costs and to increase the applications of SPECT studies [40]. Additionally, technological advances in hardware are expected to enable new quantitative capabilities in SPECT imaging [41,42].

### 2.2. Advancements in Software Techniques

Advanced software techniques have been implemented and incorporated in recent SPECT and SPECT/CT systems. These techniques have the ability to recover image resolution and to limit image noise [10,43]. Moreover, they have limited applications, which are mostly in cardiac imaging [44,45]. Most recently, improvements in software techniques have focused on reconstructions algorithms, data correction methods, and quantitative methods [1,46].

#### 2.2.1. Reconstruction Algorithms

Over the past decade, the introduction of SPECT/CT has coincided with a significant increase in computing power and iterative reconstruction algorithms, resulting in noticeable enhancements in SPECT image quality [1]. Furthermore, the utilisation of iterative algorithms, particularly the maximum likelihood expectation maximisation (MLEM) and the subsequent development of ordered-subsets expectation maximisation (OSEM), has yielded improved noise control compared with filtered back-projection methods [46]. However, a limitation of these techniques has been the amplification of image noise and the spatial variability of the point spread functions. Consequently, methods have been proposed to address spatial blurring as part of the reconstruction process. These approaches generally involve modelling the distance-dependent resolution within the projection aspect of iterative algorithms [46,47].

Recently, resolution-recovery or noise reduction algorithms have been incorporated into software packages by the majority of SPECT camera manufacturers [10]. Additionally, reconstruction algorithms such as Astonish (Philips Healthcare), Flash3D (Siemens Healthineers), Evolution (GE Healthcare), and Wide-Beam Reconstruction (UltraSPECT) have been developed for conventional SPECT cameras [10,47,48]. Notably, new ultrafast camera designs incorporate versions of these resolution-recovery and noise reduction techniques to further enhance the image spatial and contrast resolution while lowering the counting noise [10,48].

The Astonish reconstruction technique is a fast SPECT reconstruction algorithm that integrates enhancements targeting the primary elements influencing the quality of SPECT images [47,49]. Additionally, it is based on the OSEM reconstruction method, which incorporates noise reduction techniques into the iterative process and models the depth-dependent resolution of projection data [49]. Corrections for photon scatter, photon attenuation, and changes in spatial resolution are also included. Similarly, attenuation correction and resolution recovery are incorporated into the reconstruction process [48]. Moreover, in this reconstruction algorithm, the resolution-recovery correction can be performed with or without attenuation correction [47,49]. Furthermore, it smooths the measured and estimated projection data internally throughout the reconstruction process as part of a noise-reduction technique. By increasing the signal-to-noise ratio, Astonish reconstruction produces images with remarkable quality, faster speed, and improved accuracy [47,48]. As a result, the Astonish algorithm offers significant improvements in both image resolution and noise compared with conventional OSEM techniques [10]. The Astonish reconstruction shortens the MPI acquisition time without affecting the image quality.

The Flash3D method incorporates fast iterative OSEM reconstruction with 3D resolution recovery [48]. It corrects the detector response [47] and compensates for attenuation and scatter. Moreover, Flash3D has been used to create SPECT cardiac acquisition protocols (Cardio-Flash), which can reduce acquisition times by 33% to 50% when compared with conventional procedures that use FBP reconstruction [10,48,50]. Joel and co-authors [51] optimised IQ.SPECT and compared it with conventional MPI and echocardiography on 17 patients. Their study concluded that IQ.SPECT showed no significant differences to conventional SPECT in terms of the perfusion score and the estimation of the ejection fraction on gated images while shortening the scanning time by at least a factor of 3.

The Evolution technique represents a modification of the OSEM algorithm, introducing resolution recovery under the name “Evolution for Cardiac” or OSEM-RR (for OSEM resolution recovery). This method entails integrating the collimator and detector response functions into an iterative reconstruction algorithm [10,47,48]. Within the OSEM-RR model, a basic collimator geometric response function is included for round-hole shaped collimators. The evolution reconstruction algorithm addresses and compensates for various parameters such as the collimator hole, crystal thickness, collimator-to-detector gap, and projection-angle-specific centre of rotation-to-collimator face distance [48]. On the other hand, certain acquisition parameters (such as object-to-collimator distance) are obtained straight from the raw projection data, while specific collimator data are saved as lookup tables. To limit the noise amplification inherent in resolution recovery during iterative reconstruction, OSEM-RR also utilises noise suppression, much like other optimal reconstruction techniques. This could potentially reduce the creation of hot spots in the final image [10,48].

Wide-Beam Reconstruction (WBR), like other modified reconstruction algorithms discussed previously, models the physics and geometry of the emission and detection process and aims for resolution recovery [50,52]. Additionally, during iterative reconstruction, it corrects reconstructed voxels and takes into account the distance between the detector and the patient. The WBR approach provides information regarding the geometry of the collimator, such as the dimensions and shape of holes or septa thickness. This solution is able to reconstruct data from the latest gamma cameras with a standard collimator design. Moreover, WBR reduces the acquisition time, improves the image quality, and reduces patient radiation exposure [53,54].

#### 2.2.2. Data Correction Methods

The combination of attenuation, scattered radiation, poor spatial resolution, and noise degrades the quality of SPECT images. Consequently, the information acquired by SPECT systems does not accurately represent the projection information required for precise reconstruction. The detection of scattered gamma rays in SPECT affects both the image quality and the quantitative accuracy [11,55]. Moreover, traditional scatter correction methods have limitations such as accuracy and noise issues and hence have not been widely applied. Consequently, several new and improved scatter correction methods have been developed over the past decade, with increased complexity and computational time [56].

Various approaches to estimating scatter exist, including by direct measurement in additional energy windows or through modelling. Moreover, scatter estimation during iterative image reconstruction significantly benefits from increased computing speed. Recent resolution-recovery-based iterative reconstruction algorithms integrate scatter correction to enhance image quality by more accurately compensating for scattered photons. This process is computationally intensive, involving complex calculations that account for scatter effects at each iteration. With advancements in computing speed, these algorithms can perform scatter corrections more efficiently, reducing the time required for image reconstruction [54,56].

Recently, with an increase in computer capacity and computational speed, new data correction methods have continued to develop. In myocardial perfusion imaging, scatter correction is achieved by subtracting the counts acquired in off-peak energy windows [57,58]. This easy-to-compute strategy, however, makes images noisier. Hence, model-based scatter correction is used, which demonstrates an improvement in contrast over subtraction procedures by 10% to 20% [56]. Additionally, complex SPECT corrections are used in radionuclide dosimetry research and brain imaging [59,60].

#### 2.2.3. Quantitative Methods

Quantification is one of the key benefits of nuclear medicine imaging. Differentiating anomalies (such disease, infection, spinal fusion, and joint degeneration) from predicted physiology and accidental uptake should be the ultimate diagnostic goal of quantitative SPECT. Recently, quantitative SPECT has moved from relative and semi-quantitative metrics to absolute quantification in terms of activity concentration and normalised uptake using the standard uptake value (SUV) [61]. More recently, SPECT quantification in terms of kBq/cc or SUV has become more common due to the needs of radionuclide therapy. Therefore, software manufacturers have taken on this challenge and made such quantification easier and more accurate [61,62]. Software manufacturers have also made significant efforts towards establishing SPECT & SPECT/CT imaging as a quantitative tool by incorporating specific camera calibration and resolution-recovery-based reconstruction techniques such as xSPECT™ and SUV SPECT™ quantification [61,62].

xSPECT technology (Siemens Healthineers, Germany) fully integrates SPECT and CT data during image reconstruction, providing sharp clinical details and accurate measurements of disease conditions. xSPECT includes high-resolution xSPECT bone imaging, which aids in distinguishing bone metastases from surrounding tissue and accurately localising infection and injury [62,63]. It utilises the xSPECT Quant™ software to monitor disease progression over time, standardising uptake values for reliable disease detection and assessment of the therapy response [63]. SUV-SPECT (Hermes Medical Solutions™) offers absolute quantification of radiotracer uptake by enabling the conversion of voxel counts to an activity concentration (kBq/cc) and, thus, to quantitative SUV for SPECT [64].

Software manufacturers are poised to persist in refining reconstruction algorithms for various SPECT applications, with a particular emphasis on MPI [45,47], thus augmenting the clinical efficacy of SPECT. Moreover, enhancements in quantitative accuracy for bone and cancer therapy are anticipated across multiple vendors [61,63]. Companies such as GE Healthcare, Hermes Medical Solutions, Medical Image Merge (MIM), Osirix, and Siemens Healthineers will introduce a range of software packages equipped with advanced quantitative tools. These dedicated applications will empower end users to assess radiotracer uptake in specific volumes of interest through user-friendly graphical interfaces [64].

## 3. Advances in SPECT Animal Imaging

Animal imaging research mostly employs rodent models that are too small to be imaged adequately using clinical imaging systems. The major limitations of animal imaging include low spatial resolution, low sensitivity, and poor tissue contrast [65]. The most recent advances in preclinical imaging research focused on overcoming these limitations and enhancing the overall imaging performance by introducing specific refinements and alterations to the instrumentation [66].

Recent advances in SPECT animal imaging systems, in addition to what was presented in Section 2.1.1, include pinhole collimation to achieve better spatial resolution by means of pinhole magnification. Multiple pinholes or other multiple collimation apertures have been used to enhance the imaging sensitivity [9]. Different recent crystal materials such as CsI(Tl), CsI(Na) or LaBr3(Ce), described in great detail in Section 2.1.1, have been introduced to further increase the resolution and sensitivity. Recent animal SPECT systems are capable of detecting minute (<10^−10^ molar) radiotracer concentrations (0.1 nanomole) with submillimetre spatial resolution (0.5–0.7 mm) in vivo [67,68]. Some animal SPECT systems incorporate semiconductor detector materials (CdTe or CdZnTe (CZT)), which offer higher energy discrimination efficiency, specifically for low-energy radionuclides and dual isotope applications [9,69].

Compact SPECT animal imaging systems (called also micro-SPECT systems) that are equipped with these new technologies are widely used in several preclinical research areas for assessing myocardial perfusion, necrosis or angiogenesis in cardiovascular research [70,71]; for detecting gene expression and metastases or molecular pathways of tumour formation in oncology [72,73]; and in brain disorders, such as in research on Parkinson’s and Alzheimer’s diseases [74,75]; as well for the development of new drugs [76].

Small animal SPECT imaging research, due to the size of the imaged animals, the organs, and tissues to be evaluated, the spatial resolution necessary to detect functional or structural changes, and the total data acquisition duration, has benefited greatly from the combination of highly sensitive functional information (micro-SPECT) with high-resolution modalities (micro-CT and/or micro-MRI) techniques. This combination further improves the signal-to-noise ratio, contrast-to-noise-ratio, and provides a better localisation of signals with good temporal resolution and minimal amounts of radiopharmaceuticals. These hybrid animal imaging systems have become a major cornerstone in preclinical research and modern drug development [66].

## 4. Artificial Intelligence in SPECT and SPECT/CT Imaging 

In recent years, artificial intelligence (AI) has undergone rapid evolution, and its application in medical specialties, including nuclear medicine and healthcare, has effectively transitioned from theory to practice [77,78]. AI techniques present new opportunities in nuclear medicine thanks to the significant increase in computing power and the availability of vast digital archives to manage storage (big data). These techniques are being implemented and validated at local hospitals to ensure that they align with effective practices that aid health professionals in routine clinical service provisions. Overall, AI and its subset of machine learning (ML) techniques have assumed various roles in nuclear medicine, such as patient setup, dosimetry, quality assurance, and pre-reporting tasks [77]. ML is also utilised in medical imaging for outcome prediction, image analysis, and image reconstruction [79], thereby reducing the physician workload, patient waiting time, administrated activity, and overall imaging time in nuclear medicine [77].

AI-based applications in SPECT and SPECT/CT focus on automating repetitive and time-consuming tasks [77,80]. Some of the AI applications, including endocardial border tracking software and polar plots, are widely employed in nuclear cardiology as valuable prognostic markers [80,81]. By automating cardiac SPECT diagnosis, AI has shown great potential in enhancing the diagnosis and prediction of coronary artery diseases [82,83]. Another notable example is the use of ML-based 3D automated lung applications, which assist physicians in contouring lung nodules and performing accurate quantification before making decisions regarding surgery [84,85].

The recent development of deep learning (DL) approaches has paved the way for more accurate SPECT image reconstruction compared with conventional methods. For instance, SPECTNet was the first DL network developed for SPECT image reconstruction, demonstrating lower sensitivity to noise compared with OSEM-based methods and enabling accurate SPECT image reconstruction [86]. A future direction in SPECT and SPECT/CT techniques involves the establishment of more registries like REFINE SPECT to provide extensive databases of clinical and imaging features. These databases are instrumental in developing and fully validating DL-based methods in SPECT imaging [87]. It is anticipated that the continuous integration of AI into SPECT systems will not replace nuclear imaging experts but will instead offer enhanced diagnostic and predictive capabilities, thereby maximising patient outcome and improving service productivity [88,89]. Most recent studies outline key open research problems and offer insights into the application of machine learning and deep learning algorithms for several clinical applications, especially in brain disorders, cardiovascular disease, and cancer as well as several methodological issues such as the optimisation of image reconstruction, reduction of radiation doses, as well as creation scatter correction and attenuation maps from emission data [90,91,92,93,94,95,96].

Despite the potential of AI, demonstrated through findings reported in recent published papers, its translation into routine practice still faces several challenges, and further essential developments are still required. There appears to be a significant gap between end-user expectations and the use of AI in current clinical practice. This has been attributed to several factors, including complexities of the healthcare data structure, suitability of ML and DL models, as well as the ability to standardise big-data platforms that house text data. In addition, the reasoning process utilised to achieve the desired outcomes has led to concerns around transparency and the explainability of the AI model outcomes and the potential bias in AI-based methodologies, particularly in the case of deep learning algorithms used for image reconstruction and image analysis [97].

Multidisciplinary approaches are required to combine the efforts from clinicians, medical and radiation oncologists, radiation therapists, medical physicists, data scientists, technologists, and decision-makers in designing, developing, evaluating, and fully validating real-world AI applications [98]. A critical step to further this would also require strict ethical and legal frameworks, standardised SPECT and SPECT/CT imaging protocols, universally applicable data-restricting frameworks, and external validation. Updating higher education curriculums and specialised training programs by incorporating AI concepts, methods, and applications is another crucial element to consider [99].

Guidelines and best practices for developing, training, testing, monitoring AI techniques are being developed, and it could take a while to see AI applications fully adopted clinically and demonstrate their full potential in the effective management of diseases and heath economics [100,101]. Insights into translating and implementing AI techniques into routine clinical practice have also recently been debated in international scientific meetings [102,103].

## 5. Clinical Implications of Recent Advances in SPECT and SPECT/CT

The following sections present the clinical impact of SPECT and SPECT/CT imaging, using clinical cases performed at our institution in cardiology, oncology, musculoskeletal, neurology and infectious disease cases.

### 5.1. Cardiology

**Figure 4:**
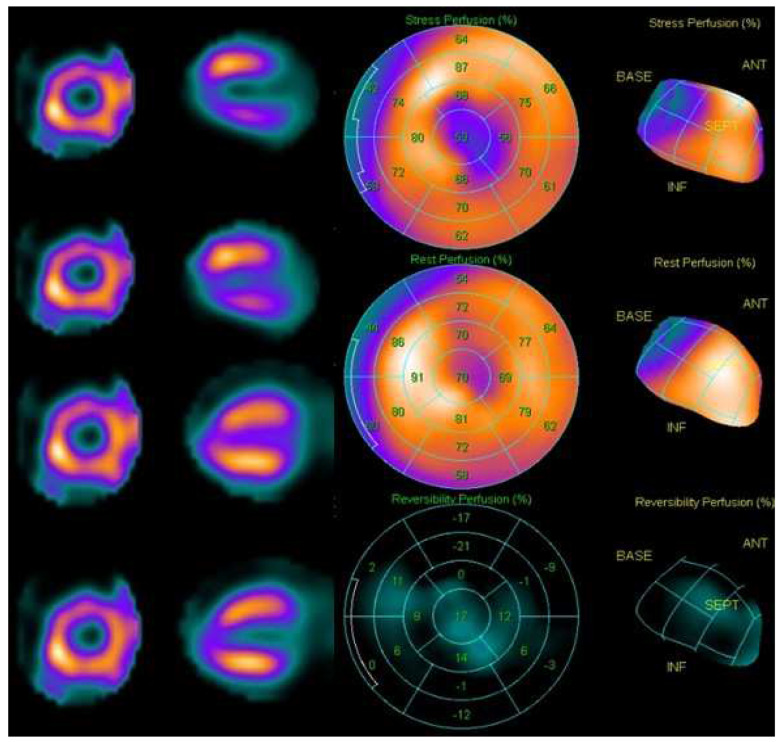
Short and horizontal long-axis SPECT images of stress (first and second rows in the first and second columns) and rest (third and fourth rows in the first and second columns) myocardial perfusions. The stress and rest myocardial perfusion scans show partial reversible perfusion defects in the apex, apico-anterior, apico-lateral and apico-inferior segments. The polar maps and rendered images of stress, rest, and irreversible perfusion in the third column (top, middle, and bottom), respectively, illustrate the hypo-perfused areas and their perfusion scores using the 16-segment model.

SPECT in nuclear cardiology has been an incomparable imaging modality for many years. The introduction of combined SPECT/CT systems has added new dimensions by providing anatomical information in addition to functional information. CT-based attenuation correction provides a significant advantage in reducing attenuation artefacts and hence easing and enhancing the quality of clinical reporting. Figure 4 below shows the case of a 57-year-old female as a known case of hypertension, dyslipidaemia, and obesity, being evaluated for chest pain. Stress and rest myocardial perfusion scans were performed with IQ.SPECT on 2 separate days to look for inducible ischemia. 

The scans show partial reversible perfusion defects in the apex, apico-anterior, apico-lateral and apico-inferior segments as depicted by the hypoperfused areas in the myocardium on the transverse and short-axis images.

### 5.2. Oncology

SPECT/CT has established itself as a powerful diagnostic tool for the accurate localisation of radiotracer distributions in nuclear medicine imaging, especially in oncology and cancer thyroid patients. It helps to accurately localise the iodine uptake in iodine whole body scans which, in turn, leads to correct risk stratification and staging of the disease.

**Figure 5:**
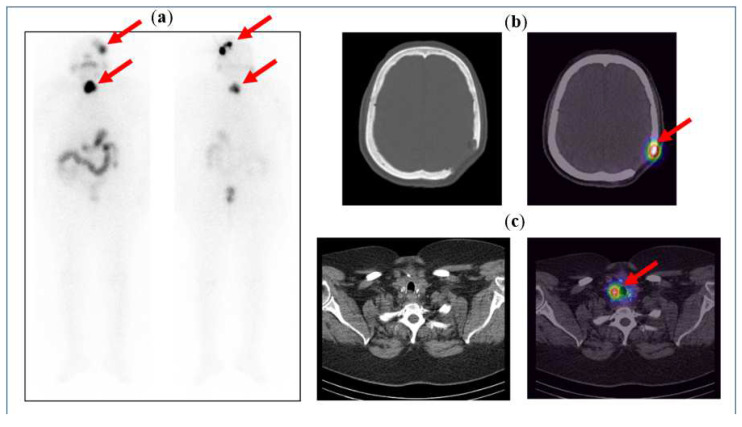
(**a**) Anterior and posterior whole-body images of the post-therapy iodine scan showing iodine uptake in the neck and the left side of the skull. Physiological tracer distribution is seen in the salivary glands (as pointed out by the arrows), stomach, bowel loops, and urinary bladder. (**b**) CT and fused SPECT/CT images of the skull showing an osteolytic lesion with tracer uptake along the post-operative margin in the left parietal bone. (**c**) CT and fused SPECT/CT images of the neck clearly show tracer uptake in the residual tissue in the neck (seen as hot spots on the image on the right).

Figure 5 below illustrates the case of a 71-year-old female, known to have follicular thyroid carcinoma and skull bone metastases, after a total thyroidectomy with neck dissection and post-excision of the skull bone metastases. The patient was given a high dose of 5.5 GBq radioactive iodine (RAI) therapy, and the post-therapy scan shows iodine uptake in the neck, which is localised to the residual thyroid tissue on the SPECT/CT images, and iodine uptake in the left parietal bone, localising the residual tumour at the post-operative margins.

### 5.3. Musculoskeletal

Musculoskeletal (MSK) imaging, especially planar whole-body bone scans, have been widely used in nuclear medicine for many years. Its high sensitivity and ability to depict bone changes before other imaging modalities have made it important in the diagnosis of a wide range of bone diseases. SPECT/CT has provided new dimensions to bone scanning in terms of the better localisation and characterisation of MSK diseases. 

**Figure 6:**
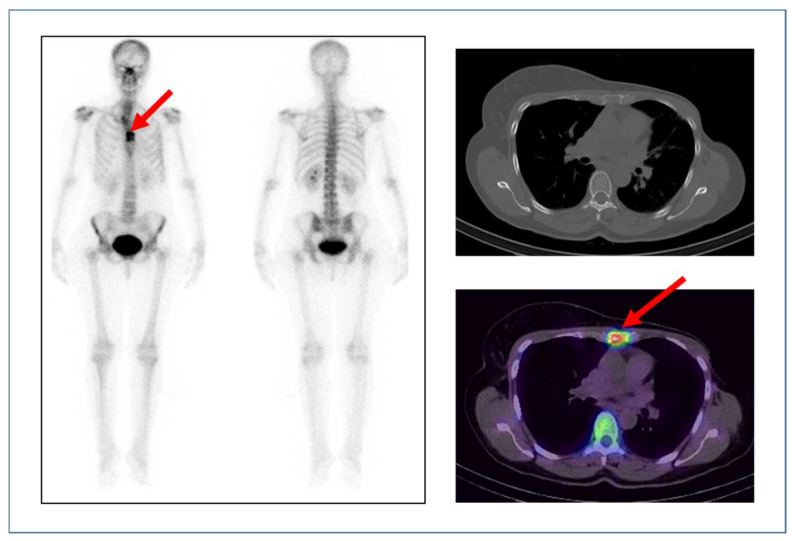
Anterior and posterior whole-body bone images show abnormal radiotracer uptake in the sternum as pointed out by the arrow (**left**). CT and fused SPECT/CT (**right top**) and (**right bottom**) images of the chest show abnormal tracer uptake (seen as a hot spot on the image) in the sternum at the site of the osteolytic lesion as indicated by the arrow.

Figure 6 presents the case of a 41-year-old female at a follow-up for left breast cancer. The scan was performed post-left modified radical mastectomy (MRM) and post-chemotherapy with tamoxifen. The patient presented with anterior chest pain; a bone scan was performed for the evaluation of chest pain. The bone scan shows abnormal osteoblastic activity in the sternum (on whole-body planar images) which localises to an osteolytic lesion seen on SPECT/CT fused images, suggesting the diagnosis of bone metastasis.

### 5.4. Neurology

The introduction of hybrid imaging with SPECT/CT in nuclear medicine imaging has revolutionised the acquisition, interpretation, and diagnosis of neurological disorders leading to the improved detection and localisation of lesions, which hence impacts positively on patient care. An example of that is the use of SPECT/CT for the detection and localisation of cerebrospinal fluid (CSF) leakage using SPECT/CT radionuclide cisternography.

**Figure 7:**
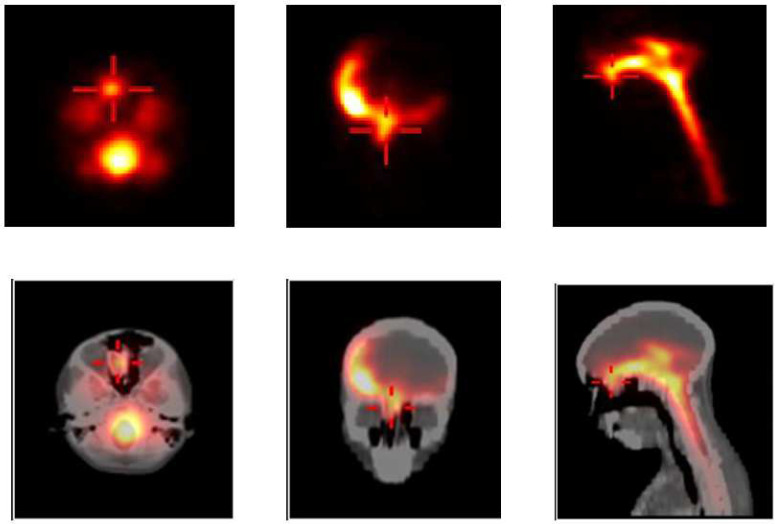
Planar static images (**top row left**: axial view; **top row middle**: coronal view; **top row right**: sagittal view) show an abnormal focal uptake in the frontal skull region as depicted by the cross. **Bottom row**: SPECT-CT views localising the uptake to the right ethmoid bone.

Figure 7 illustrates the clinical case of an 11-year-old boy with a 3-month history of rhinorrhoea following a head trauma. His clinical evaluation and skull X-rays were normal, and he was referred to the nuclear medicine department for radionuclide cisternography to check for CSF leakage. Under aseptic technique, 125 MBq of Tc99m-DTPA was injected intrathecally, and static as well as SPECT/CT images were acquired. SPECT/CT was able to detect a CSF leakage and accurately localised it to the right ethmoid bone. These findings were confirmed at surgery.

### 5.5. Infection

The diagnosis of infection can be a huge challenge for clinicians. The detection and accurate localisation of the focus of infection has improved with the use of modern high-sensitivity hybrid SPECT/CT systems. This is especially important in patients with fever of unknown origin, suspected bone or soft tissue infection, and vascular graft infection, as well as in the assessment of the diabetic foot.

**Figure 8:**
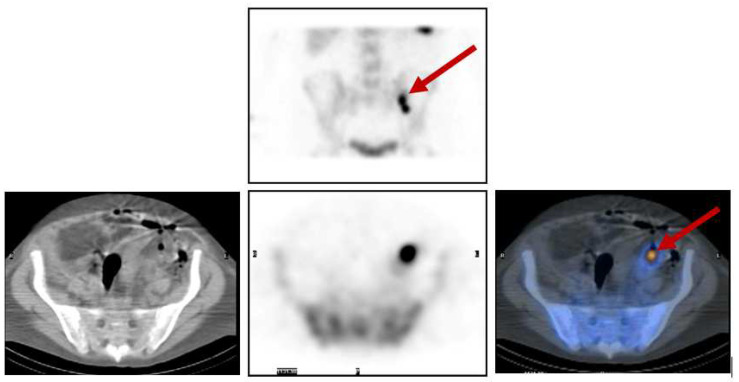
**Top row**: Planar static image (anterior view). **Bottom row**: SPECT/CT axial images (CT slice—left; SPECT slice—middle; and fused images—right) that localise the abnormal uptake to an intra-abdominal abscess in the peritoneum.

Figure 8 shows the clinical case of a 33-year-old male with fever of unknown origin, where all previous investigations had failed to detect the source of infection. Tc-99m white blood cells (WBC) whole-body scans and SPECT/CT detected and localized, respectively, an intra-abdominal abscess, as indicated by the arrows.

## 6. Conclusions

The reviewed literature in this paper suggests that the fast growth and technical advances in SPECT and SPECT/CT are securing them a more competitive position among diagnostic imaging techniques. Comprehensive comparison studies using the latest SPECT hardware and software techniques with other non-ionising modalities, such as magnetic resonance imaging and ultrasound imaging, is one way of preserving the competitive position of SPECT and SPECT/CT. Respective research areas could also include developing more accurate and quantitative SPECT images with refinements in corrections for scattered radiation, attenuation, and spatial resolution losses. Moreover, optimisation of reconstruction techniques to include count statistics and specific system calibration data will offer better spatial resolution and quantification of pertinent clinical parameters at an early stage of the disease. Software techniques, which could be used, for example, to separate the myocardium tracer uptake from other organs and to ease segmentation, would be useful for the diagnosis and follow-up of cardiac diseases. The use of machine and deep learning techniques for diagnosis and the prediction of therapy outcomes will largely benefit from the improved image quality offered by commercially available techniques (e.g., segmentation of brain and cardiac anatomical structures and extraction of radiomic features). Further advances in SPECT and SPECT/CT imaging technology are expected to improve clinical outcomes and ensure patient-centred, sustainable, and cost-effective models of care.

## Data Availability

All data generated or analysed during this study are included in the manuscript (use of images for illustrations).

## References

[1] Seo Y., Mari C., Hasegawa B.H. (2008). Technological development and advances in single-photon emission computed tomography/computed tomography. Semin. Nucl. Med..

[2] Sharir T., Slomka P.J., Berman D.S. (2010). Solid-state SPECT technology: Fast and furious. J. Nucl. Cardiol..

[3] Madsen M.T. (2007). Recent advances in SPECT imaging. J. Nucl. Med..

[4] Hutton B.F. (2014). The origins of SPECT and SPECT/CT. Eur. J. Nucl. Med. Mol. Imaging.

[5] Ritt P., Kuwert T. (2020). Quantitative SPECT/CT-Technique and Clinical Applications. Recent Results Cancer Res..

[6] Anzola L.K., Hernandez N., Rodriguez L.F., Sanguino G., Martinez E., Lopez R., Moreno S., Prill R., Mut F., Rasch H. (2023). The role of SPECT/CT in painful, noninfected knees after knee arthroplasty: A systematic review and meta-analysis—A diagnostic accuracy review. J. Orthop. Surg. Res..

[7] Hutton B.F., Erlandsson K., Thielemans K. (2018). Advances in clinical molecular imaging instrumentation. Clin. Transl. Imaging.

[8] Melcher C.L. (2005). Perspectives on the future development of new scintillators. Nucl. Instrum. Methods Phys. Res. A.

[9] Peterson T.E., Furenlid L.R. (2011). SPECT detectors: The Anger Camera and beyond. Phys. Med. Biol..

[10] Garcia E.V., Faber T.L., Esteves F.P. (2011). Cardiac dedicated ultrafast SPECT cameras: New designs and clinical implications. J. Nucl. Med..

[11] Wernick M.N., Aarsvold J.N. (2004). Emission Tomography: The Fundamentals of PET and SPECT.

[12] Renker D. (2007). New trends on photodetectors. Nucl. Instrum. Methods Phys. Res. A.

[13] Li C., Huang W., Gao L., Wang H., Hu L., Chen T., Zhang H. (2020). Recent advances in solution-processed photodetectors based on inorganic and hybrid photo-active materials. Nanoscale.

[14] Loudos G.K., Nikita K.S., Uzunoglu N.K., Giokaris N.D., Papanicolas C.N., Archimandritis S.C., Maintas D. (2003). Improving spatial resolution in SPECT with the combination of PSPMT based detector and iterative reconstruction algorithms. Comput. Med. Imaging Graph..

[15] Pani R., Pellegrini A., Soluri A., Vincentis G.D., Filippi S., Pergola A., Domenico G.D., Guerra A.D., Scopinaro F. (1997). New generation position-sensitive PMT for nuclear medicine imaging. Nucl. Instrum. Methods Phys. Res. A.

[16] Pani R., Soluri A., Scafe R., Pergola A., Pellegrini R., Trotta G., Scopinaro F., Vincentis G.D. (1999). Multi-PSPMT scintillation camera. IEEE Trans. Nucl. Sci..

[17] Stapels C.J., Squillante M.R., Lawrence W.G., Augustine F.L., Christian J.F. (2007). CMOS-based avalanche photodiodes for direct particle detection. Nucl. Instrum. Methods Phys. Res. A.

[18] Funk T., Després P., Barber W.C., Shah K.S., Hasegawa B.H. (2006). A multipinhole small animal SPECT system with submillimeter spatial resolution. Med. Phys..

[19] Occhipinti M., Busca P., Butt A.D., Cozzi G., Fiorini C., Perali I., Ferri A., Gola A., Piemonte C. A compact SiPM photodetector array for SPECT applications. Proceedings of the 2014 IEEE Nuclear Science Symposium and Medical Imaging Conference (NSS/MIC).

[20] Moehrs S., Guerra A.D., Herbert D.J., Mandelkern M.A. (2006). A detector head design for small-animal PET with silicon photomultipliers (SiPM). Phys. Med. Biol..

[21] Jaszczak R.J., Greer K.L., Coleman R.E. (1988). SPECT using a specially designed cone beam collimator. J. Nucl. Med..

[22] Caobelli F., Kaiser S.R., Thackeray J.T., Bengel F.M., Chieregato M., Soffientini A., Guerra U.P. (2014). IQ SPECT allows a significant reduction in administered dose and acquisition time for myocardial perfusion imaging: Evidence from a phantom study. J. Nucl. Med..

[23] Gremillet E., Agostini D. (2016). How to use cardiac IQ• SPECT routinely? An overview of tips and tricks from practical experience to the literature. Eur. J. Nucl. Med. Mol. Imaging.

[24] Havel M., Kolacek M., Kaminek M., Dedek V., Kraft O., Sirucek P. (2014). Myocardial perfusion imaging parameters: IQ-SPECT and conventional SPET system comparison. Hell. J. Nucl. Med..

[25] Nakajima K., Okuda K., Momose M., Matsuo S., Kondo C., Sarai M., Vija A.H. (2017). IQ· SPECT technology and its clinical applications using multicenter normal databases. Ann. Nucl. Med..

[26] Garcia E.V., Faber T.L. (2009). New trends in camera and software technology in nuclear cardiology. Cardiology clinics. Cardiol. Clin..

[27] Slomka P.J., Patton J.A., Berman D.S., Germano G. (2009). Advances in technical aspects of myocardial perfusion SPECT imaging. J. Nucl. Cardiol..

[28] Matsuo S., Nakajima K., Onoguchi M., Wakabayash H., Okuda K., Kinuya S. (2015). Nuclear myocardial perfusion imaging using thallium-201 with a novel multifocal collimator SPECT/CT: IQ-SPECT versus conventional protocols in normal subjects. Ann. Nucl. Med..

[29] Pirich C., Keinrath P., Barth G., Rendl G., Rettenbacher L., Rodrigues M. (2017). Diagnostic accuracy and functional parameters of myocardial perfusion scintigraphy using accelerated cardiac acquisition with IQ SPECT technique in comparison to conventional imaging. Q. J. Nucl. Med. Mol. Imaging.

[30] Rajaram R., Bhattacharya M., Ding X., Malmin R., Rempel T.D., Vija A.H., Zeintl J. Tomographic performance characteristics of the IQ.SPECT system. Proceedings of the 2011 IEEE Nuclear Science Symposium Conference Record.

[31] Zaman M.U., Hashmi I., Fatima N. (2010). Recent developments and future prospects of SPECT myocardial perfusion imaging. Ann. Nucl. Med..

[32] Buechel R.R., Herzog B.A., Husmann L., Burger I.A., Pazhenkottil A.P., Treyer V., Kaufmann P.A. (2010). Ultrafast nuclear myocardial perfusion imaging on a new gamma camera with semiconductor detector technique: First clinical validation. Eur. J. Nucl. Med. Mol. Imaging.

[33] Erlandsson K., Kacperski K., Van G.D., Hutton B.F. (2009). Performance evaluation of D-SPECT: A novel SPECT system for nuclear cardiology. Phys. Med. Biol..

[34] Gambhir S.S., Berman D.S., Ziffer J., Gramberg D.V., Hutton B.F. (2009). A novel high-sensitivity rapid-acquisition single-photon cardiac imaging camera. J. Nucl. Med..

[35] Sharir T., Ben-Haim S., Merzon K., Prochorov V., Dickman D., Ben-Haim S., Berman D.S. (2008). High-speed myocardial perfusion imaging: Initial clinical comparison with conventional dual detector anger camera imaging, JACC. Cardiovasc. Imaging.

[36] Pretorius P.H., Liu C., Fan P., Peterson M., Ljungberg M. (2015). Monte Carlo simulations of the GE discovery alcyone CZT SPECT systems. IEEE Trans. Nucl. Sci..

[37] Patton J.A., Slomka P.J., Germano G., Berman D.S. (2007). Recent technologic advances in nuclear cardiology. J. Nucl. Cardiol..

[38] Imbert L., Chevalier E., Claudin M., Karcher G., Verger A., Paycha F., Marie P.Y. (2019). A one-shot whole-body bone SPECT may be recorded in less than 20 minutes with the high-sensitivity Veriton® CZT-camera. J. Nucl. Med..

[39] Imbert L., Jurczak J., Perrin M., Karcher G., Marie P.Y., Verger A. (2019). Image quality of brain SPECT recorded with the whole-body Veriton CZT camera and a focal brain configuration of detectors, as compared with conventional SPECT and PET systems. J. Nucl. Med..

[40] Heller S.L., Goodwin P.N. (1987). SPECT instrumentation: Performance, lesion detection, and recent innovations. In Seminars in nuclear medicine. Semin. Nucl. Med..

[41] Beller G.A. (2007). Quantitative nuclear cardiology and future directions for SPECT imaging. J. Nucl. Cardiol..

[42] Zaidi H. (2006). Recent developments and future trends in nuclear medicine instrumentation. Z. Med. Phys..

[43] Ljungberg M., Pretorius P.H. (2018). SPECT/CT: An update on technological developments and clinical applications. Br. J. Radiol..

[44] Beller G.A. (2010). Recent advances and future trends in multimodality cardiac imaging. Heart Lung Circ..

[45] Abbott B.G., Case J.A., Dorbala S., Einstein A.J., Galt J.R., Pagnanelli R., Bullock-Palmer R.P., Soman P., Wells R.G. (2018). Contemporary Cardiac SPECT Imaging—Innovations and Best Practices: An Information Statement from the American Society of Nuclear Cardiology. Circ. Cardiovasc. Imaging.

[46] Ritt P., Sanders J., Kuwert T. (2014). SPECT/CT technology, Clin. Transl. Imaging.

[47] Knoll P., Kotalova D., Köchle G., Kuzelka I., Minear G., Mirzaei S., Bergmann H. (2012). Comparison of advanced iterative reconstruction methods for SPECT/CT. Z. Med. Phys..

[48] O’Mahoney E., Murray I. (2013). Evaluation of a matched filter resolution recovery reconstruction algorithm for SPECT-CT imaging. Nucl. Med. Commun..

[49] Daou D., Pointurier I., Coaguila C., Vilain D., Benada A.B., Lebtahi R., Fourme T., Slama M., Le Guludec D. (2003). Performance of OSEM and depth-dependent resolution recovery algorithms for the evaluation of global left ventricular function in 201Tl gated myocardial perfusion SPECT. J. Nucl. Med..

[50] Vija A.H., Hawman E.G., Engdahl J.C. (2003). Analysis of a SPECT OSEM reconstruction method with 3D beam modeling and optional attenuation correction: Phantom studies. Nucl. Sci. Symp. Conf. Record..

[51] Joel E., Bouchareb Y., Haroon A., Luqman M., Newell M., Jan H. Optimisation of IQ.SPECT in Myocardial Perfusion Imaging–Comparison with Conventional SPECT and Echocardiography Imaging. Proceedings of the Nuclear Science Symposium and Medical Imaging Conference.

[52] DePuey G., Gadiraju R., Anstett F. (2007). OSEM and WBR half-time gated myocardial perfusion SPECT: A comparison to filtered backprojection. J. Nucl. Med..

[53] DePuey E.G., Bommireddipalli S., Clark J., Leykekhman A., Thompson L.B., Friedman M. (2011). A comparison of the image quality of full-time myocardial perfusion SPECT vs wide beam reconstruction half-time and half-dose SPECT. J. Nucl. Cardiol..

[54] Borges-Neto S., Pagnanelli R.A., Shaw L.K., Honeycutt E., Shwartz S.C., Adams G.L., Coleman R.E. (2007). Clinical results of a novel wide beam reconstruction method for shortening scan time of Tc-99m cardiac SPECT perfusion studies. J. Nucl. Cardiol..

[55] Zaidi H., Koral K.F. (2004). Scatter modelling and compensation in emission tomography. Eur. J. Nucl. Med. Mol. Imaging.

[56] Hutton B.F., Buvat I., Beckman F.J. (2011). Review and current status of SPECT scatter correction. Phys. Med. Biol..

[57] Dewaraja Y.K., Wilderman S.J., Ljungberg M., Koral K.F., Zasadny K., Kaminiski M.S. (2005). Accurate dosimetry in 131I radionuclide therapy using patient-specific, 3-dimensional methods for SPECT reconstruction and absorbed dose calculation. Journal of Nuclear Medicine. J. Nucl. Med..

[58] He B., Frey E.C. (2006). Comparison of conventional, model-based quantitative planar, and quantitative SPECT image processing methods for organ activity estimation using In-111 agents. Phys. Med. Biol..

[59] Barrett H.H., Abbey C.K., Clarkson E. (1998). Objective assessment of image quality. III. ROC metrics, ideal observers, and likelihood-generating functions. J. Opt. Soc. Am. A Opt. Image Sci. Vis..

[60] Frey E.C., Gilland K.L., Tsui B.M. (2002). Application of task-based measures of image quality to optimization and evaluation of three-dimensional reconstruction-based compensation methods in myocardial perfusion SPECT. IEEE. Trans. Med. Imaging.

[61] Dickson J., Ross J., Vöö S. (2019). Quantitative SPECT: The time is now. EJNMMI Phys..

[62] Armstrong I.S., Hoffmann S.A. (2016). Activity concentration measurements using a conjugate gradient (Siemens xSPECT) reconstruction algorithm in SPECT/CT. Nucl. Med. Commun..

[63] Ma J., Vija A.H. Evaluation of quantitation accuracy for xSPECT. Proceedings of the 2015 IEEE Nuclear Science Symposium and Medical Imaging Conference (NSS/MIC).

[64] Ross J.C., Vilić D., Sanderson T., Vöö S., Dickson J. (2019). Does quantification have a role to play in the future of bone SPECT?. Eur. J. Hybrid Imaging.

[65] Zanzonico P.P., Kiessling F., Pichler B.J. (2011). Invasive imaging for supporting basic research. Small Animal Imaging.

[66] Lauber D.T., Fülöp A., Kovács T., Szigeti K., Máthé D., Szijártó A. (2017). State of the art in vivo imaging techniques for laboratory animals. Lab. Anim..

[67] Meikle S.R., Kench P., Kassiou M., Banati R.B. (2005). Small animal SPECT and its place in the matrix of molecular imaging technologies. Phys. Med. Biol..

[68] Vastenhouw B., Beekman F. (2007). Submillimeter total-body murine imaging with U-SPECT-I. J. Nucl. Med..

[69] Franc B.L., Acton P.D., Mari C., Hasegawa B.H. (2008). Small-animal SPECT and SPECT/CT: Important tools for preclinical investigation. J. Nucl. Med..

[70] Liu Z., Kastis G.A., Stevenson G.D., Barrett H.H., Furenlid L.R., Kupinski M.A., Patton D.D., Wilson D.W. (2002). Quantitative analysis of acute myocardial infarct in rat hearts with Ischemia reperfusion using a high-resolution stationary SPECT system. J. Nucl. Med..

[71] Meoli D.F., Sadeghi M.M., Krassilnikova S., Bourke B.N., Giordano F.J., Dione D.P., Su H., Edwards D.S., Liu S., Harris T.D. (2004). Noninvasive imaging of myocardial angiogenesis following experimental myocardial infarction. J. Clin. Investig..

[72] Hanahan D., Weinberg R.A. (2000). The hallmarks of cancer. Cell.

[73] Ponsky L., Cherullo E., Starkey R., Nelson D., Neumann D., Zippe C. (2001). Evaluation of preoperative ProstaScint scans in the prediction of nodal disease. Prostate Cancer Prostatic Dis..

[74] Sharma S., Ebadi M. (2008). SPECT neuroimaging in translational research of CNS disorders. Neurochem. Int..

[75] Kung M.P., Hou C., Zhuang Z.P., Cross A.J., Maier D.L., Kung H.F. (2004). Characterization of IMPY as a potential imaging agent for b-amyloid plaques in double transgenic PSAPP mice. Eur. J. Nucl. Med. Mol. Imaging.

[76] Rudin M. (2009). Noninvasive structural, functional, and molecular imaging in drug development. Curr. Opin. Chem. Biol..

[77] Aktolun C. (2019). Artificial intelligence and radiomics in nuclear medicine: Potentials and challenges. Eur. J. Nucl. Med. Mol. Imaging.

[78] Davenport T., Kalakota R. (2019). The potential for artificial intelligence in healthcare. Future Health J..

[79] Choi H. (2018). Deep learning in nuclear medicine and molecular imaging: Current perspectives and future directions. Nucl. Med. Mol. Imaging.

[80] Shameer K., Johnson K.W., Glicksberg B.S., Dudley J.T., Sengupta P.P. (2018). Machine learning in cardiovascular medicine: Are we there yet?. Heart.

[81] Kurgan L.A., Cios K.J., Tadeusiewicz R., Ogiela M., Goodenday L.S. (2001). Knowledge discovery approach to automated cardiac SPECT diagnosis. Artif. Intell. Med..

[82] Yoneyama H., Nakajima K., Taki J., Wakabayashi H., Matsuo S., Konishi T., Okuda K., Shibutani T., Onoguchi M., Kinuya S. (2019). Ability of artificial intelligence to diagnose coronary artery stenosis using hybrid images of coronary computed tomography angiography and myocardial perfusion SPECT. Eur. J. Hybrid. Imaging.

[83] Apostolopoulos I.D., Papandrianos N.I., Feleki A., Moustakidis S., Papageorgiou E.I. (2023). Deep learning-enhanced nuclear medicine SPECT imaging applied to cardiac studies. EJNMMI Phys..

[84] Whiston B.A., Growth S.S., Maddaus M.A. (2007). Surgical assessment and intraoperative management of mediastinal lymph nodes in non-small cell lung cancer. Ann. Thorac. Surg..

[85] Pena D.M., Luo S., Abdelgader A. (2016). Auto diagnostics of lung nodules using minimal characteristics extraction technique. Diagnostics.

[86] Shao W., Du Y. (2019). SPECT Image Reconstruction by Deep Learning Using a Two-Step Training Method. J. Nucl. Med..

[87] Slomka P.J., Betancur J., Liang J.X., Otaki Y., Hu L.-H., Sharir T., Dorbala S., Di Carli M., Fish M.B., Ruddy T.D. (2018). Rationale and design of the REgistry of Fast Myocardial Perfusion Imaging with NExt generation SPECT (REFINE SPECT). J. Nucl. Cardiol..

[88] Jimenez-Mesa C., Arco J.E., Martinez-Murcia F.J., Suckling J., Ramirez J., Gorriz J.M. (2023). Applications of machine learning and deep learning in SPECT and PET imaging: General overview, challenges and future prospects. Pharmacol. Res..

[89] Arabi H., Allaf A.A., Sanaat A., Shiri I., Zaidi H. (2021). The promise of artificial intelligence and deep learning in PET and SPECT imaging. Phys. Med..

[90] Boyle J., Gaudet V.C., Black S.E., Vasdev N., Rosa-Neto P., Zukotynski K.A. (2021). Artificial intelligence for molecular neuroimaging. Ann. Transl. Med..

[91] Madan N., Lucas J., Akhter N., Collier P., Cheng F., Guha A., Zhang L., Sharma A., Hamid A., Ndiokho I. (2022). Artificial intelligence and imaging: Opportunities in cardio-oncology. Am. Heart J. Plus.

[92] Papachristou K., Panagiotidis E., Makridou A., Kalathas T., Masganis V., Paschali A., Aliberti M., Chatzipavlidou V. (2023). Artificial intelligence in Nuclear Medicine Physics and Imaging. Hell. J. Nucl. Med..

[93] Shao W., Rowe S.P., Du Y. (2021). Artificial intelligence in single photon emission computed tomography (SPECT) imaging: A narrative review. Ann. Transl. Med..

[94] Petmezas G., Papageorgiou V.E., Vassilikos V., Pagourelias E., Tsaklidis G., Katsaggelos A.K., Maglaveras N. (2024). Recent Advancements and Applications of Deep Learning in Heart Failure: A Systematic Review. Comput. Biol. Med..

[95] Tran K.A., Kondrashova O., Bradley A., Williams E.D., Pearson J.V., Waddell N. (2021). Deep learning in cancer diagnosis, prognosis and treatment selection. Genome Med..

[96] Triantafyllidis A., Kondylakis H., Katehakis D., Kouroubali A., Koumakis L., Marias K., Alexiadis A., Votis K., Tzovaras D. (2022). Deep Learning in mHealth for Cardiovascular Disease, Diabetes, and Cancer: Systematic Review. JMIR Mhealth Uhealth.

[97] Pesapane F., Codari M., Sardanelli F. (2018). Artificial intelligence in medical imaging: Threat or opportunity? Radiologists again at the forefront of innovation in medicine. Eur. Radiol. Exp..

[98] Martín-Noguerol T., Paulano-Godino F., López-Ortega R., Górriz J., Riascos R.F., Luna A. (2021). Artificial intelligence in radiology: Relevance of collaborative work between radiologists and engineers for building a multidisciplinary team. Clin. Radiol..

[99] Bouchareb Y., Delanerolle G., Raniga S., Al-Dhuhli H. Towards an Effective Deployment of Artificial Intelligence in Routine Practice: What Should Radiology and Molecular Imaging Professionals Consider?. Proceedings of the ECR 2023.

[100] Jha A.K., Bradshaw T.J., Buvat I., Hatt M., Kc P., Liu C., Obuchowski N.F., Saboury B., Slomka P.J., Sunderland J.J. (2022). Nuclear Medicine and Artificial Intelligence: Best Practices for Evaluation (the RELAINCE Guidelines). J. Nucl. Med..

[101] Bradshaw T.J., Huemann Z., Hu J., Rahmim A. (2023). A Guide to Cross-Validation for Artificial Intelligence in Medical Imaging. Radiol. Artif. Intell..

[102] Bouchareb Y., Khaniabadi P.M., Reguna S., Al-Dhuhli H. (2021). Radiomics and artificial intelligence: How medical physicists can help their translation into radiology, molecular imaging and radiation therapy routine clinical practice?. Phys. Medica.

[103] European Society of Radiology (ESR) (2019). What the radiologist should know about artificial Intelligence—An ESR white paper. Insights Imaging.

